# Comparative Genome of GK and Wistar Rats Reveals Genetic Basis of Type 2 Diabetes

**DOI:** 10.1371/journal.pone.0141859

**Published:** 2015-11-03

**Authors:** Tiancheng Liu, Hong Li, Guohui Ding, Zhen Wang, Yunqin Chen, Lei Liu, Yuanyuan Li, Yixue Li

**Affiliations:** 1 Key Lab of Systems Biology, Institute of Biochemistry and Cell Biology, Shanghai Institutes for Biological Sciences, Chinese Academy of Sciences, Shanghai, China; 2 Shanghai Center for Bioinformation Technology, Shanghai, China; University of Heidelberg, GERMANY

## Abstract

The Goto-Kakizaki (GK) rat, which has been developed by repeated inbreeding of glucose-intolerant Wistar rats, is the most widely studied rat model for Type 2 diabetes (T2D). However, the detailed genetic background of T2D phenotype in GK rats is still largely unknown. We report a survey of T2D susceptible variations based on high-quality whole genome sequencing of GK and Wistar rats, which have generated a list of GK-specific variations (228 structural variations, 2660 CNV amplification and 2834 CNV deletion, 1796 protein affecting SNVs or indels) by comparative genome analysis and identified 192 potential T2D-associated genes. The genes with variants are further refined with prior knowledge and public resource including variant polymorphism of rat strains, protein-protein interactions and differential gene expression. Finally we have identified 15 genetic mutant genes which include seven known T2D related genes (*Tnfrsf1b*, *Scg5*, *Fgb*, *Sell*, *Dpp4*, *Icam1*, and *Pkd2l1*) and eight high-confidence new candidate genes (*Ldlr*, *Ccl2*, *Erbb3*, *Akr1b1*, *Pik3c2a*, *Cd5*, *Eef2k*, and *Cpd*). Our result reveals that the T2D phenotype may be caused by the accumulation of multiple variations in GK rat, and that the mutated genes may affect biological functions including adipocytokine signaling, glycerolipid metabolism, PPAR signaling, T cell receptor signaling and insulin signaling pathways. We present the genomic difference between two closely related rat strains (GK and Wistar) and narrow down the scope of susceptible loci. It also requires further experimental study to understand and validate the relationship between our candidate variants and T2D phenotype. Our findings highlight the importance of sequenced-based comparative genomics for investigating disease susceptibility loci in inbreeding animal models.

## Background

Type 2 diabetes (T2D), also known as non-insulin-dependent diabetes is characterized by defects in both insulin secretion and utilization and accounts for about 90% of the 346 million diabetic cases around the world [1]. Both environmental and genetic factors contribute to the etiology and progression of T2D [2, 3]. In the last two decades, significant efforts, ranging from traditional candidate gene mapping to genome-wide association studies (GWAS), have identified nearly 120 T2D susceptibility genes in different human population [3–24]. However, the precise molecular pathogenesis of this heterogeneous disease remains poorly characterized. Therefore more T2D-related genes are expected to be uncovered.

The Goto-Kakizaki (GK) rat, a non-obese animal model of T2D, was developed by repeated inbreeding of glucose-intolerant Wistar rats [25]. GK rats suffer from reduced beta-cell mass with insulin resistance. Therefore, these model rats provide an ideal model system to search for T2D susceptible genes/loci to enhance our understanding of the etiology and pathogenesis of the disease [26, 27]. Several quantitative trait locus (QTL) analyses on this model have already identified a number of genomic loci harboring susceptible variants [28–30].

As large-scale changes in the genome such as copy number variations (CNVs) have been linked to dozens of human diseases [31–35], we previously conducted a genome-wide screen for CNVs between GK (T2D model) and Wistar rat (wild type) using comparative genomic hybridization (CGH) array. A set of T2D-associated CNV regions with the total length of about 36 Mb, including several novel T2D susceptibility loci which contain 16 protein-coding genes (*Il18r1*, *Cyp4a3*, *Sult2a1*, *Sult2a2*, *Sult2al1*, *Nos2*, *Pstpip1*, *Ugt2b*, *Uxs1*, *RT1-A1*, *RT1-A3*, *RT1-Db1*, *RT1-N1*, *RT1-N3*, *RT1-O*, and *RT1-S2*) and two microRNA genes (*rno-mir-30b* and *rno-mir-30d*), were identified [36].

The draft genome of the Brown Norway (BN) rat, covering around 92% of the genome, was released in 2004 [37], and was the third complete mammalian genome to be deciphered. Limitations in obtaining extensive genetic data have been largely overcome by the development and maturation of next-generation sequencing (NGS) technologies, which have significantly improved the throughput with reduced costs. Whole genome sequencing has become an alternative approach to identify genes involved in disease. For example, comparing the genomic sequence of spontaneously hypertensive rats (SHR) and the BN reference genome, Atanur et al., identified a number of candidate loci that may be involved in the development of hypertension [38].

**Fig 1:**
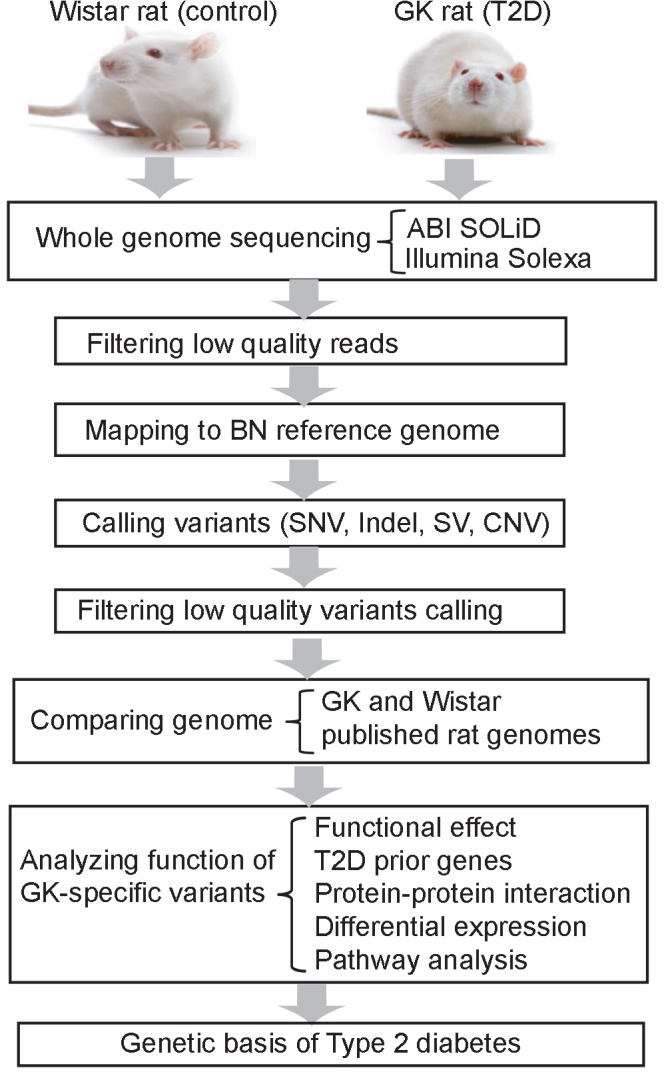
Pipeline for whole-genome sequencing and comparative analysis between GK and Wistar rats.

We conducted whole genome sequencing and compared the genomic sequence differences between Wistar and GK rats (Fig 1). As a result we generated a list of GK-specific variations including 228 structural variations, 2660 CNV amplifications, 2834 CNV deletions, and 1796 protein affecting SNVs or indels. Comparing these variations with known rat genome variations and known human T2D-associated genes, we obtained 192 candidate genes including 15 with high confidence that may be associated with the T2D phenotype observed in GK rats. These genes are involved in multiple pathways, suggesting that multiple interacting biological networks may be involved in the GK T2D phenotype. We expected this work facilitates the understanding of the molecular processes involved in the development of T2D.

## Results

### Sequencing and mapping

We sequenced the genome of GK/Slac and Wistar/Slac using both Illumina Solexa and ABI SOLiD platforms. Sequencing reads from these two platforms were combined in the subsequent analyses to gain higher sequence coverage and depth. In total, we obtained 100.4 Gb for the GK/Slac and 85.6 Gb for the Wistar/Slac rat which represent an average sequencing depth of 35.9X for GK/Slac and 30.5X for Wistar/Slac (S1 File). About 50.45% (43.27%) SOLiD reads and 32.13% (18.68%) Solexa reads of GK/Slac (Wistar/Slac showed in parentheses) were evaluated as low quality by a strict quality control procedure (S2 File). These low quality reads were filtered before sequence mapping. The remained reads were mapped to BN reference genome, obtaining more than 99% coverage (99.40% and 99.35% genome were covered by at least one read in GK/Slac and Wistar/Slac sequencing, respectively; 93.02% and 93.65% genome were covered by at least five reads).

In order to get accurate variant calls, we reassessed the quality of the mapping results through the GATK workflow [39]. After these steps, 0.42% (0.58%) of the Solexa reads, and 6.05% (4.87%) of the SOLiD reads were removed from GK/Slac (Wistar/Slac) mapping results, respectively (S3 File). In total we aligned 34.06 Gb (34.70 Gb) from GK/Slac (Wistar/Slac) sequencing data to the BN reference rat genome which corresponded to 13.27X (13.52X) coverage of high-quality reads for GK/Slac (Wistar/Slac) [38].

### Variant calling

Sequence variants identified in GK/Slac and Wistar/Slac include single nucleotide variant (SNV), small insertion and deletion (indel), structural variation (SV), and copy number variation (CNV). In total, we identified 3,471,498 (3,194,675) raw SNVs and 492,731 (517,005) raw small indels for GK/Slac (Wistar/Slac). These variants were further filtered by sequence coverage, strand bias, and error-enriched regions (see Method for detailed description) which resulted in 3,049,694 (2,727,649) high-quality SNVs and 476,841 (487,315) high-quality indels for GK/Slac (Wistar/Slac) (S4 File). Among them 2,927,627 (2,623,154) were homozygous SNVs in GK/Slac (Wistar/Slac). The percentage of homozygous SNVs was 95.99% (96.16%), consistent with the expected level for the inbred strain. There were 2,066,576 (1,843,133) transitions and 983,003 (884,403) transversion sequence changes in GK/Slac (Wistar/Slac). The ratio of transition to transversion ratio (Ti/Tv) is 2.10 (2.08), which is comparable to the Ti/Tv ratio of ~2.0–2.1 observed in human genomic sequence dataset [40].

Among all SNVs, 2,695,132 (66.8%) of them were present in the dbSNP database (http://rgd.mcw.edu/pub/data_release/GFF/SNPS/DbSNP/). Others may be strain-specific or private SNVs that were not covered by previous studies.

To evaluate the accuracy of variant calling, we compared the primary results to a public dataset from the STAR project [41]. This dataset includes genotypes for 20,238 SNVs across 167 distinct inbred rat strains including 10 GK and 6 Wistar strains. There were 7368 (4000) positions that were mutant in all GK (Wistar) strains, and 2491 (1372) positions that were not polymorphic in all GK (Wistar) strains. We checked our SNV calling results against these positions and calculated sensitivity and specificity. For GK/Slac strain, we called 6984 SNVs among the 7368 positions (94.79% sensitivity) and 2489 out of the 2491 non-polymorphic positions (99.9% specificity). For Wistar/Slac strain, we called 3319 SNVs among the 4000 positions (82.97% sensitivity) and 1104 out of the 1372 non-polymorphic positions (80.47% specificity).

### Comparative genome analysis

**Fig 2:**
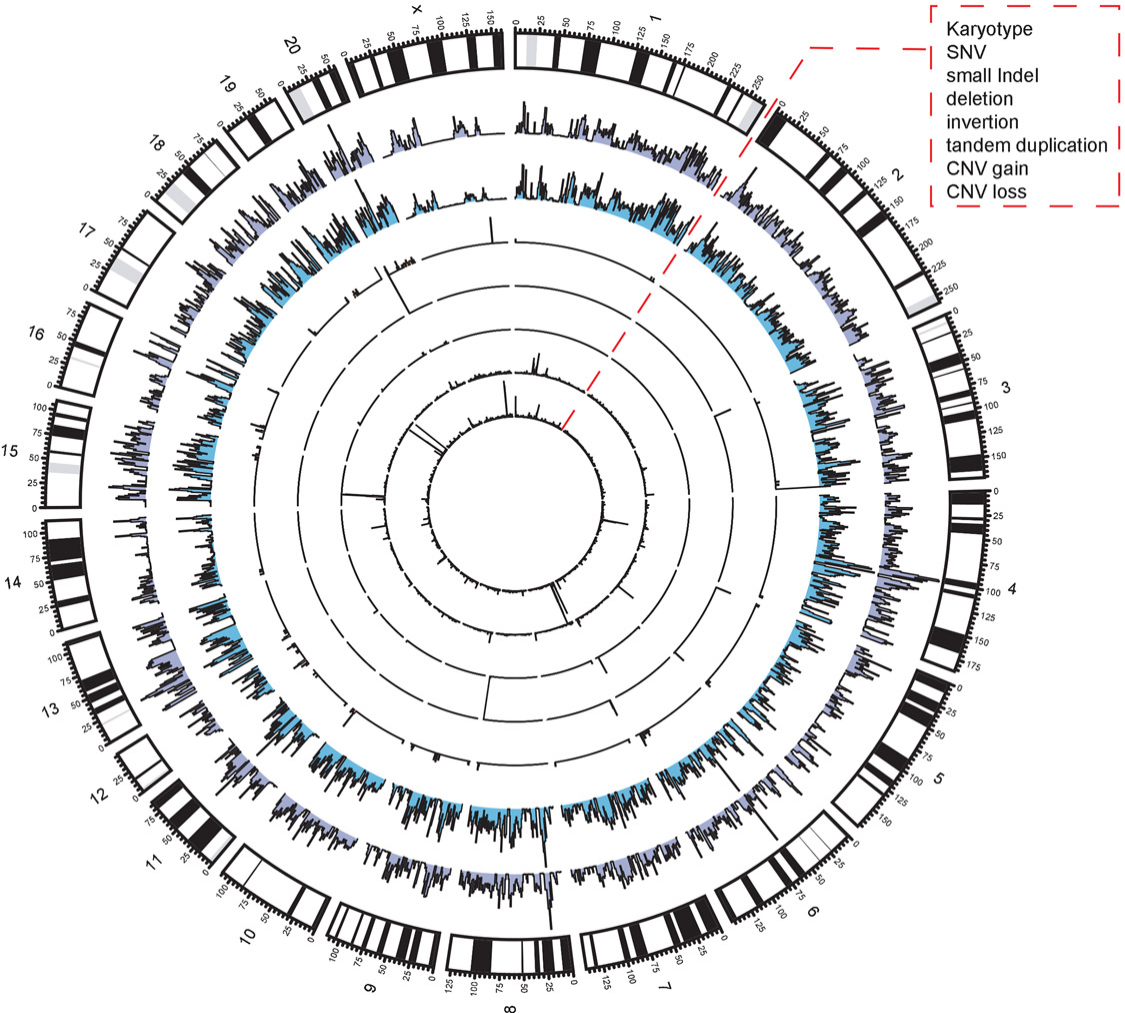
Densities for 7 kinds of GK/Slac specific variants on the rat genome. Each tiny bar stand for variants density normalized in 1 Mb genomic segments (see Methods), which was plotted on the circular chromosomes by CIRCOS (http://http://circos.ca/). Layers from outside to inside represent for rat kayrotype and the density of SNV, small indel, large deletion, inversion, tandem duplication, CNV gain, and CNV loss.

Since the GK rat was obtained by selective inbreeding of Wistar rats, their specific genetic changes from Wistar should indicate the cause of type 2 diabetic phenotypes observed. Therefore, GK/Slac specific variants were determined by comparing variants in GK/Slac with Wistar/Slac. All GK/Slac specific variants were shown on a circular chromosome map (Fig 2).

**Fig 3:**
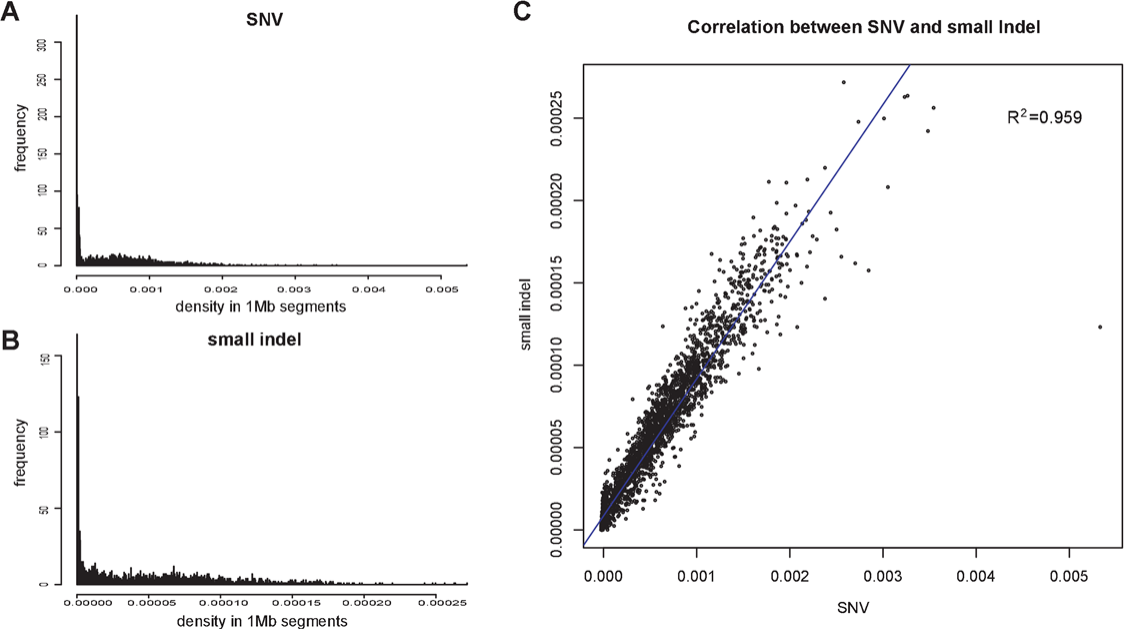
Density and distribution of SNVs and small indels. (A). Distribution of SNV density in 1Mb segments. (B) Distribution of small-indel density in 1Mb segments. (C) Correlation between the distribution of SNVs and small indels.

There were 1,354,739 GK/Slac specific SNVs and 134,554 GK/Slac specific indels. The density of GK/Slac specific SNV/indel was calculated in each 1Mb segment, and their distribution was plotted in Fig 3A and 3B. Most genomic regions were relatively conserved with extremely low SNV density (0–0.0001) and regions with median SNV density (0.0001–0.001) were evenly distributed. When the SNV density increased, the frequency decreased smoothly (0.001–0.002). A long tail indicated the existence of extremely high SNV density (>0.002) regions.

The distribution of indel density was similar to the SNV distribution (Fig 3A and 3B). We calculated the Pearson correlation coefficient between SNV density and indel density in each 1Mb genomic segment. The result showed that the density frequency of GK/Slac specific SNVs and indels were positively correlated (R^2^ = 0.959, Fig 3C). This indicated some SNVs and indels tend to co-localize at highly mutated regions of the genome (S5 File). As expected, there were regions in the genome that showed no or very few differences between GK/Slac and Wistar/Slac strain, termed variants deserts. Examples included chromosome 1 (20Mb-21Mb) and chromosome X (53Mb-54Mb).

Besides SNV and small indels, we also compared large SVs and CNVs between GK/Slac and Wistar/Slac. We identified 228 GK/Slac specific SVs, including 174 deletions, 12 inversions, 36 tandem duplications and 6 translocations. Based on sequence coverage, we predicted 2660 CNV amplified regions and 2834 CNV deleted regions between the GK/Slac and Wistar/Slac rat genomes. To validate our CNV calling, we compared the CNV candidates with a set of 116 CNVs identified by CGH array data from our previous work [36]. Out of 58 CNV gain regions identified by array, we successfully identified 38 in the sequencing results. The sequencing results also identified 31 out of the 58 CNV loss regions identified by array.

### Identification of potential T2D candidate mutations specifically presented in the GK rat

We were interesting in the GK/Slac specific variants that might contribute to the development of type 2 diabetes phenotype in GK/Slac. We mapped GK/Slac specific SVs and CNVs to regions containing functional transcripts. For SVs, 26 genes were affected, including 18 olfactory receptor genes (ORs) (S7C File). 75 genes were associated with CNVs, among them 24 were OR genes (S7D File). The OR gene family is the largest superfamily in mammalian genome. There are 1,493 OR genes in the rat and 19.5% may be pseudo-genes [42]. OR genes are frequently clustered in regions with a large number of retrotransposons or around subtelomeric regions [43] [44], which tend to exhibit a high rate of mutation. Therefore, we thought GK/Slac specific variants in ORs were background mutations rather than causal of the T2D phenotype. Besides the OR genes, other SV or CNV affected genes were randomly distributed with no enrichment in T2D related pathways and no literature evidence of either direct or indirect association between these genes and T2D.

**Fig 4:**
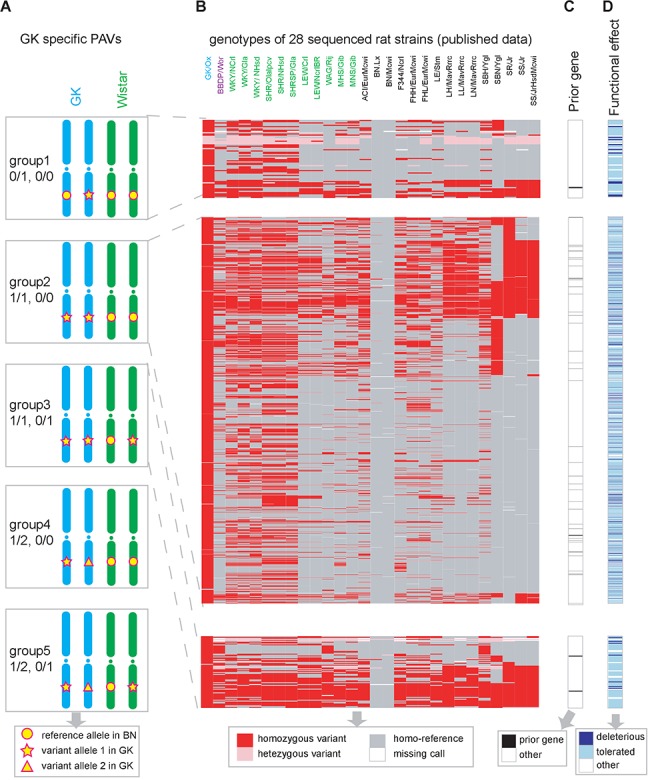
Analysis of GK/Slac specific protein affecting SNVs. (A) Variants were classified into five groups based on their genotypes in GK/Slac and Wistar/Slac. As shown in the bottom legend, circles stand for the original reference allele whereas stars and triangles represent two different mutant alleles. Taken group 1 as an example, variants is heterozygous in GK/Slac that have one mutant allele and one reference allele, while it is homozygous-reference in Wistar/Slac. Almost all variants are in group1, group2, and group3. (B) Genotype profiling for 1762 GK/Slac specific SNVs in 28 previous sequenced rat strains. GK/Ox and GK/Slac are GK strains which came from different geographical locations. BBDP is a type 1 diabetic model, another 11 Wistar derived rats are labeled by green. (C) T2D related prior genes. (D) Functional effect of nonsynonymous SNVs predicted by SIFT.

Next we investigated potential T2D candidate variants from GK/Slac-specific SNVs and indels. We divided SNP/indels into five groups to illustrate their genotype patterns in GK/Slac and Wistar/Slac (Fig 4A). Group1 (0/1, 0/0) contained sites that were heterozygous variant in GK/Slac and homozygous reference in Wistar/Slac; Group2 (1/1, 0/0) contained sites that were homozygous variant in GK/Slac and homozygous reference in Wistar/Slac; Group3 (1/1, 0/1) contained sites that were homozygous variant in GK/Slac and heterozygous variant in Wistar/Slac; Group4 (1/2, 0/0) was similar with Group2 and Group5 (1/2, 0/1) was similar with Group3, which were rare sites with two mutant alleles. Among 1,354,739 GK/Slac specific SNVs, group 1 to 3 accounted for the majority of SNVs with 3.6%, 92.9% and 3.5%, respectively (Table 1). Like SNVs, among 134,554 GK/Slac specific indels, proportion of group1 to group3 were 5.0%, 81.9% and 13.1%, respectively. In summary, most SNV and indel variants belonged to groups 1, 2 and 3, and only few allele sites had the complicated allele composition in group 4 and 5 which was consistent with the low probability of *de novo* production of two rare alleles in the lab inbreeding strain. Group 2 accounted for a large proportion that was concordant with the high homozygosity rate of inbred laboratory rat. Next we annotated the functional effect of GK/Slac specific SNVs/indels by ANNOVAR [45]. Table 2 showed the number of SNPs/indels in each genotype group and functional class. Variants had potential to interrupt the protein functions were called protein affecting variants (PAVs), including nonsynonymous, stopgain, stoploss, splicing, frameshift indels and exonic ncRNA. We detected 1796 PAVs, including 1762 SNVs and 34 indels (S7AB File).

**Table 1:** Five different genotype of GK/Slac specific SNVs and indels.

GK/Slac specific	Total	Group1 (0/1, 0/0)	Group2 (1/1, 0/0)	Group3 (1/1, 0/1)	Group4 (1/2, 0/0)	Group5 (1/2, 0/1)
**SNVs**	1354739	48573	1258423	47735	2	6
**indels**	134554	6731	110171	17647	1	4

**Table 2:** Functional annotation of GK specific variants. There are 1796 GK/Slac specific protein affecting variants, including 1762 SNVs and 34 indels. Values in table are the number of SNVs in each function class and values in parentheses are the number of indels.

	Annotation	Total	Group1 (0/1, 0/0)	Group2 (1/1, 0/0)	Group3 (1/1, 0/1)	
**Protein affecting variants**	**Nonsynonymous**	1721	106	1526		89
	**Stopgain**	14	2	12		0
	**Stoploss**	1	1	0		0
	**RNA splicing**	8 (5)	1 (1)	7 (2)		0 (2)
	**ncRNA_exonic**	18 (1)	1	17 (1)		0
	**Frameshift deletion**	(18)	(4)	(13)		(1)
	**Frameshift insertion**	(10)	(2)	(8)		(0)
	**Synonymous**	3485	154	3173		158
**Other variants**	**UTR**	4484 (467)	214 (23)	4076 (393)		194 (51)
	**Intronic**	303876 (32349)	10279 (1523)	283225 (26523)		10372 (4303)
	**Up/Down-stream**	13145 (1456)	503 (68)	12084 (1207)		558 (181)
	**Intergenic**	1027253 (100220)	37282 (5109)	953648 (82009)		36323 (13102)

**Fig 5:**
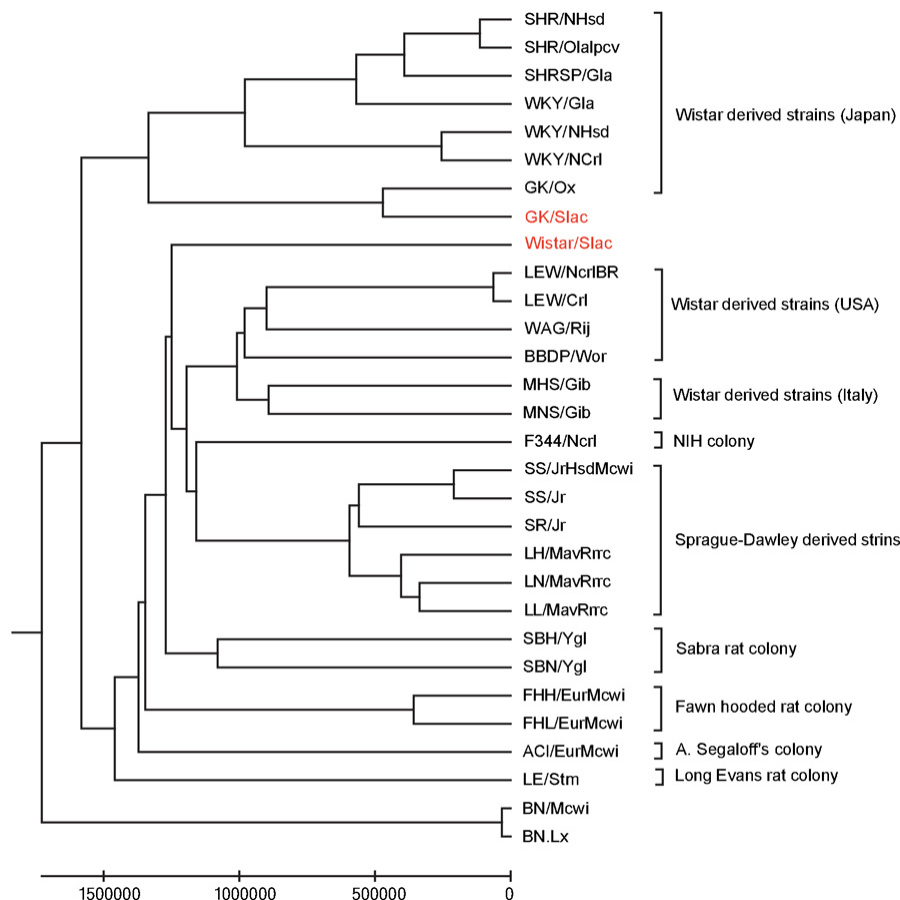
Phylogenetic Tree of GK/Slac, Wistar/Slac and other sequenced rat strains. Whole genome sequencing of GK/Slac and Wistar/Slac were performed in this study. Whole-genome SNPs of other strains were obtained from Atanur et.al. [46]. Distance between all possible pairs of strains were measured by net nucleotide substitutions [88]. The phylogenetic tree was constructed using UPGMA (unweighted pair-group method with arithmetic means) method in MEGA 6.06 package.

To further refine the above PAVs, we compared our variants with the variants of public RGD datasets. Atanur et al. reported whole-genome sequencing results of 28 laboratory rat strains[46]. Depending on these variants and ours, we plotted a phylogenetic tree for these rats (Fig 5). As the phylogenetic relationship showed, GK/Slac was close to GK/Ox, and Wistar/Slac was close to Wistar derived strains in USA. Therefore, the genetic background of GK/Slac and Wistar/Slac were more similar with 12 Wistar derived strains (SHR/NHsd, SHRSP/Gla, SHR/OlaIpcv, WKY/ NCrl, WKY/Gla, WKY/NHsd, LEW/Crl, LEW/NcrlBR, WAG/Rij, BBDP/Wor, MHS/Gib, MNS/Gib) than other rat strains, which convinced our samples and results were reliable.

In the light of the public resources of variants from different rat strains, we were able to further narrow down the mutant profile. Fig 4B, 4C and 4D showed the genotype profiles of 1762 GK/Slac specific PAVs in 28 rat strains, the overlap with T2D prior genes (S6 File), and the predicted functional effect of PAVs. To identify T2D phenotype-specific genetic changes, we further filtered the 1796 GK/Slac specific PAVs based on the genotype profile of 11 Wistar strains (except BBDP/Wor, which is a type 1 diabetic model) and 1 GK/Ox strain. Our GK specific variants, which had potential to contribute to T2D phenotype, were required to present in the GK/Ox strain but not the other 11 Wistar strains.

Considering the laboratory inbreeding process, we supposed homozygous variants in GK rat have a higher probability to account for the disease phenotype. Among the 1762 GK/Slac specific protein affecting SNVs, 300 were homozygous variants in both GK strains (GK/Slac in our report and GK/Ox strain studied by Atanur et.al. [46]), but did not present in other 11 Wistar strains. These 300 SNVs were located in 252 genes including 60 OR genes and the other 192 genes were used for further analysis (S8A File). We also checked 34 protein affecting indels. Besides 7 indels were heterozygous in GK/Slac, one homozygous indel resided in the T2D prior gene *Hif1a*, but many other rat strains also had this homozygous indel; the other 26 homozygous indels were either located in OR genes or not reported to be associated with T2D.

### Refinement of the genes with homozygous mutation based on PPI network and gene expression identify prior T2D genes and new candidates

**Fig 6:**
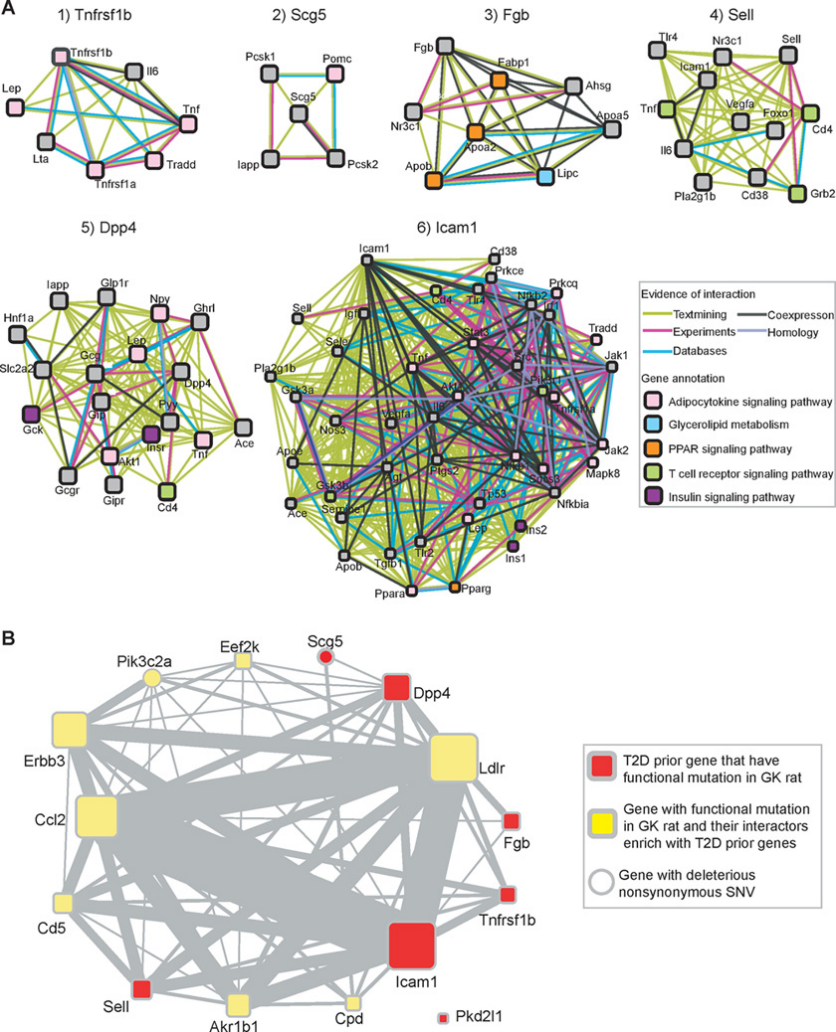
Protein-protein interaction (PPI) network for T2D candidate genes identified in GK rats. (A) PPI network for six T2D prior genes that have GK/Slac specific PAVs. Edges indicate PPI got from STRING database (only considering interaction with other T2D prior genes). Genes involved in important KEGG pathways were shown by colored boxes. (B) Relationship network among fifteen T2D candidate genes. Seven genes were T2D prior genes and have GK/Slac specific PAVs; another eight genes were enriched with T2D prior genes as PPI partners. Widths of edges were proportional to the number of shared PPI patterns.

Among 192 potential T2D associated genes, seven of them (*Tnfrsf1b*, *Scg5*, *Fgb*, *Sell*, *Dpp4*, *Icam1*, and *Pkd2l1*) were clearly reported to be T2D prior genes (see Methods for detailed description, S6 File)). As T2D phenotype was correlated with protein network dysregulation, we hypothesized T2D candidate genes were more likely to have interactions with reported T2D prior genes. We used Fisher’s test to evaluate whether their interaction partners were enriched with T2D prior genes (S8B File). There were 16 genes whose interaction partners were enriched with prior genes (adjusted p-value<0.05), among of which six genes (*Tnfrsf1b*, *Scg5*, *Fgb*, *Sell*, *Dpp4*, and *Icam1*) were known T2D prior genes. Fig 6A shows the protein-protein interaction (PPI) network between these six genes and other T2D prior genes. PPIs contained validated and predicted protein associations from STRING database and genes were annotated by KEGG pathway database. Five T2D related pathways were labeled by different colored boxes, including Adipocytokine signaling pathway, Glycerolipid metabolism, PPAR signaling pathway, T cell receptor signaling pathway and Insulin signaling pathway.

The six T2D prior genes were closely correlated with T2D phenotype according to previous investigations. Genetic variation in or near *Tnfrsf1b* might predispose clinical neuropathy, reduced glycosylated hemoglobin, and increased HDL cholesterol in type 2 diabetes patients. The latter could be part of a protective response [47]. *Tnfrsf1b* and its interacting proteins were involved in the adipocytokine signaling pathway and increased *TNF-alpha* action would protect the organism from the damage by increasing HDL cholesterol in T2D patients [47, 48]. The nonsynonymous SNV in *Scg5* (chr3: 99641204:G->C) was predicted to be deleterious (Fig 4D) by SIFT [49]. Its homologous site in mouse is annotated as “type 2 diabetes mellitus 2 in SMXA RI mice” based on QTL data in UCSC genome browser. Also *Scg5* (*SGNE1*) might impair glucose intolerance and insulin resistance [50], which was consistent with the insulin resistant phenotype of GK strain. *Fibrinogen* (*Fgb*) was described as one of the cardiovascular risk factors [51] and *Fgb* concentration was correlated with fasting insulin concentration [52]. *Fgb* was also involved in T2D related PPARγ signaling pathways [53]. *Sell* was a cell surface adhesion/homing receptor that played important roles in leukocyte-endothelial cell interactions. Although its interaction partners did not show enrichment in any T2D related pathway, previous literature had reported that *Sell* was associated with T2D-associated pathologies, such as diabetic microangiopathy [54], nephropathy [55] and diabetic retinopathy [56]. *Dpp4* was a famous drug target of T2D [57], and *Dpp4* inhibitors could ameliorate many symptom of T2D [58, 59]. PPI showed that *Dpp4* was involved in a number of biological functions (Fig 6A) [57]. *Dpp4* played a critical role in both the adipocytokine signaling pathway and insulin signaling pathway [60]. Inhibiting *Dpp4* activity increased glucose-dependent insulinotropic polypeptide and glucagon like peptide 1 induced insulin secretion [61]. T2D patients showed increased *ICAM-1* and *VCAM-1* plasma concentrations, which was thought to be related to hyperglycaemia [62]. *Pkd2l1* had been associated with T2D by GWAS study [63] and Mancusi S et al. demonstrated that the expression change of *PKD2*, which was responsible for the formation of the renal cysts and associated with early diabetes onset [64].

Some of the mutant genes were supposed to show expression changes between GK and Wistar strain. We compared the expression profile of 192 potential T2D genes in GK and Wistar rats by analyzing a public microarray dataset (GSE13271). There were 32 differentially expressed genes and 38 differential co-expressed genes in at least one tissue between GK and Wistar rat (S8B File). Among above 16 candidate genes, seven of them (*Tnfrsf1b*, *Ldlr*, *Pik3c2a*, *Sell*, *Icam1*, *Eef2k*, *Cpd*) also had significant expression changes (differentially expression or differential co-expression) between GK and Wistar. (Table 3)

**Table 3:** High-confident T2D candidate genes and their homozygous SNVs in GK rat.

Gene	Chromosome location	Nucleotide change	Protein change	SIFT prediction	Evidence of association^a^	Previous publication	Adjusted P-value^b^	Differential (co-) expression
# GK-specific homozygous variants, not in Wistar/Slac and other sequenced strains.								
Tnfrsf1b	chr5: 163680822	C->T	p.A51T	tolerated	1), 2), 3)	[47]	4.49E-2	adipose^c^
Scg5	chr3: 99641204	G->C	p.P156R	deleterious	1), 2)	[50]	1.56E-2	
Fgb	chr2: 174775040	G->T	p.L8I	tolerated	1)	[51, 52]	5.16E-3	
Sell	chr13: 79831304	C->G	p.R167G	tolerated	1), 3)	[60, 73]	5.39E-3	muscle^c^
Dpp4	chr3: 44359652	T->C	p.I20V	tolerated	1)	[57, 59, 60, 94]	2.28E-13	
Ldlr	chr8: 20844228	A->G	p.K742E	tolerated	2), 3)	[65–68]	2.73E-33	liver^d^
Ccl2	chr10: 70257779	A->C	p.E141D	tolerated	2)	[69, 70]	4.91E-22	
Erbb3	chr7: 1859081	C->T	p.R1072H	tolerated	2)	-	1.80E-18	
Akr1b1	chr4: 61654486	T->C	p.S77G	tolerated	2)	[71, 72]	8.97E-7	
Pik3c2a	chr1: 174467615	C->T	p.V598I	deleterious	2), 3)	[73]	1.14E-4	adipose^d^
Pik3c2a	chr1: 174436078	T->C	p.T1550A	tolerated	2), 3)	-	1.14E-4	adipose^d^
Cd5	chr1: 213215518	C->T	p.V445M	tolerated	2)	-	2.33E-3	
Icam1	chr8: 20050122	A->G	p.I314V	tolerated	1), 2), 3)	[62]	1.05E-23	adipose^d^
Pkd2l1	chr1: 249075571	A->T	p.T169S	-	1)	[63] [64]	1	
Pkd2l1	chr1: 249075657	G->T	p.L197F	deleterious	1)	[63] [64]	1	
Eef2k	chr1: 179746185	A->G	p.Q547R	tolerated	2), 3)	[74]	2.26E-4	muscle^d^
Cpd	chr10: 67359919	C->T	p.P490S	tolerated	2), 3)	[75]	2.23E-2	muscle^c^, adipose^c^, liver^c^

a. Additional evidence of association with T2D. 1) T2D prior genes curated from literatures. 2) Protein-protein interaction partners are enriched with T2D prior genes. 3) Differential expression or differential co-expression in GSE13271 dataset.

b. P-value for enrichment of T2D prior genes in the interaction partners. P-value was calculated by fisher test, and was adjusted by *p*.*adjust* in *R*.

c. Differential expression between GK and Wistar rats.

d. Differential co-expression between GK and Wistar rats.

Integrating prior knowledge, PPI network and differential gene expression, we finally selected 15 higher confidence T2D candidate genes with homozygous variants in GK strains (Table 3). These 15 genes were involved in multiple T2D related pathways. We counted the number of shared interaction partners between any two genes, and constructed a relationship network for 14 genes out of the 15 high-confidence T2D candidate genes (Fig 6B). Fig 6B illustrates the close relationship between 8 new genes (*Ldlr*, *Ccl2*, *Erbb3*, *Akr1b1*, *Pik3c2a*, *Cd5*, *Eef2k*, *Cpd*) and known T2D prior genes, indicating these 8 genes have strong relationships with T2D associated pathways. We manually checked their function and possible relationship to T2D in published literature. For instance, *Ldlr* had previously been shown to be associated with diabetes mellitus and its lipid phenotype [65]. A GWAS study of French population also identified *Ldlr* as a T2D risk locus [66]. *Akt2/Ldlr* double knockout mice displayed impaired glucose tolerance [67], and increased inflammation response [68]. *CCL2/C-C chemokine receptor 2* (*CCR2*) signaling was suggested to play a significant role in diabetic nephropathy and in adipose tissue inflammation during insulin resistant. Blocking *CCL2/CCR2* signaling not only improved blood glucose levels but also altered renal nephrin and *VEGF* expressions in type 2 diabetic mouse model [69]. Butcher et.al. showed that type 2 diabetic islets displayed higher *CCL2* expression than healthy islets [70]. Polymorphism of *Akr1b1* was associated with diabetic nephropathy and type 2 diabetes mellitus by two GWAS studies [71, 72]. *Pik3c2a* played a critical role in insulin secretion in β cells and down-regulation of *PI3K-C2α* might be a feature of type 2 diabetes [73]. *Eef2k* was a kinase of *Eef2* and renal cortical homogenates from db/db mice in early stage of type 2 diabetes showed decrease in *Eef2* phosphorylation and increment in *Eef2* kinase phosphorylation [74]. *Carboxypeptidase D* (*Cpd*) and *carboxypeptidase E* (*Cpe*) belonged to same family of enzymes and defects in *Cpe* could lead to β-cell stress and hyperproinsulinemia, both of which were features of type 2 diabetes in GK rat [75]. Chu KY et al. also found that *Cpd* was significantly up-regulated by elevated glucose or low doses of insulin [76].

## Conclusion

We presented a comprehensive analysis pipeline of re-sequencing study with general case-control study design. Besides identifying some prior T2d genes with mutations, we found 8 new candidate genes which required further wet-lab experimental evaluation and validation. As a bioinformatic analysis of NGS data, our workflow could be adopted in other re-sequencing study of organism with disease model.

## Discussion

Rodents have been used to model human diseases because of their similarity in genome and physiology. GK is a classic rat T2D model, which is obtained by selective inbreeding of Wistar rats. GK/Slac and Wistar/Slac rats have been bought from a commercial company (www.slaccas.com), which import rat strains from the place of production and then bred locally in China. These two strains have been widely used by Chinese investigators [77–82]. Our work provides the whole-genome sequences of GK/Slac strain and Wistar/Slac strain for the first time. This sequencing dataset will be very useful for the researchers who use these two strains as study objects. It is also an important complement to the Rat Genome Database (RGD) [83], which include the international sequencing resources of different rat strains.

Comparing the whole genome sequence of T2D phenotypic GK rats with the corresponding Wistar rats provides insights into the genomic evolution of GK during the laboratory selective inbreeding for developing the insulin resistant T2D phenotype. In the light of sequencing technology, the genetic difference between T2D GK and control Wistar rats is easy to identify. Many years of selective inbreeding in the laboratory makes these genetic variants are correlated to a consistent phenotype. Such advantages make the GK rat an ideal model to discover T2D causative genes. Here we studied the genomes of GK and Wistar rats by both sequencing and computational strategies. We have combined two sequencing platforms with different read lengths and insert-sizes. The reads are processed with stringent quality control to obtain accurate high-quality variants. In order to identify the causal variants of T2D phenotype, we used Wistar strains as background to screen GK specific variants, which not only are required to present consistently homozygous in both our and public GK rat samples, but also are absent in either our Wistar sample or any Wistar derived samples from the public databases. Then we selected high-confidence affected genes by integrating T2D prior knowledge, protein interaction and gene expression data. Finally we got fifteen genes with homozygous SNVs in GK rats and their functions are related with T2D phenotype. The integration of public resources and prior knowledge can increase the power of detection and narrow down the scale of candidates. Our data mining approach would inspire similar bioinformatic studies for disease animal model.

Our analysis focus on variants that affect protein sequences so that variants in the intergenic or intronic regions are ignored due to the lack of function annotations for these regions in rat genome. The understanding of noncoding regions may be improved by more studies on translational regulation and evolutionary conserved regions. We also observed that GK strains and Wistar strains coming from different laboratories have slightly genetic difference (Fig 4B, Fig 5), showing it is important to use biological repeats even for inbreed organisms. With decreased sequencing cost and improved computational ability, it is possible to sequence more samples to increase analysis power. The whole-genome sequencing-based disease study will be extended to other disease models and our approach can be used as an example to study these disease model organisms.

## Methods

### Sample preparation

One male GK/Slac rat and one male Wistar/Slac rat were obtained from *SHANGHAI SLAC LABORATORY ANIMAL CO*. *LTD* (www.slaccas.com). The rats were anesthetized by formalin at the age of 8 weeks, and the blood was taken from the pericardia with anticoagulant. Genomic DNA was then isolated using DNeasy Blood & Tissue Kit (Qiagen, p/n69504). All animal experiments were approved by the Biomedical Research Ethics Committee of Shanghai Institutes for Biological Sciences, Chinese Academy of Sciences (IRB00005813).

### Whole genome sequencing and data preprocessing

GK/Slac and Wistar/Slac rat DNA samples were sequenced by ABI SOLiD and Illumina Solexa paired-end sequencing technologies. To increase the coverage of genome, three SOLiD sequencing libraries and one Solexa library were constructed with different read length and insert length (S1 File). All sequence reads were deposited in the European Nucleotide Archive under accession number PRJEB6678.

Read quality was assessed by per base sequence quality, per sequence quality score, per base N content and overrepresented sequences using software FastQC (http://www.bioinformatics.babraham.ac.uk/projects/fastqc/). Low quality reads were filtered by stringent criteria: 1) removing overrepresented adaptor found by FastQC using FASTX-Toolkit (http://hannonlab.cshl.edu/fastx_toolkit/), 2) removing N base and low quality base (Phred quality score was below 20), 3) removing reads that’s shorter than 15 bp or paired read was filtered.

### Mapping to reference genome

In order to combine data from two different sequencing platforms, we transferred ABI SOLiD color space encoding data and their quality file to Solexa base space encoding format (Fastq file). Then we aligned the high-quality reads to the BN rat reference genome (UCSC rn4 / Baylor HGSC Build 3.4) by Bowtie 2 with default parameters [84]. The coverage proportion of reference genome and estimated genome were calculated by the following formula:

$$\text{reference coverage proportion}=\frac{\text{number of bases that}\prime \text{s covered by at least}\;1\;\text{read}}{\text{total bases in reference genome}}$$

$$\text{genome coverage proportion}=\frac{\text{number of bases that}\prime \text{s covered by at least}\;1\;\text{read}}{\text{estimated number of bases in the genome}}$$

We further filtered the mapping results to increase the accuracy of variant calling. Firstly, we did local realignment around known indels using Smith-Waterman algorithm. Then we removed duplicate reads to reduce amplification bias. Lastly we recalibrated base quality depending on the reference genome and dbSNP information. These three main processes were done by GATK (The Genome Analysis Toolkit) [39], and PICARD TOOLS and SAMTOOLS [85] were used to sort the bam file, fix mate pair information and do format transformation, which facilitated the GATK running process.

### Variants calling and comparison

After pre-mapping and post-mapping quality control, remained bam files were used to call variants: single nucleotide variant (SNVs), small insertion and deletion (indels), structural variation (SVs), and copy number variations (CNVs).

Small indels and SNVs were called by the UnifiedGenotyper module in GATK software and filtered by following filtering criteria: minimum number of consensus is 5, minimum base quality required to consider a base for calling is 17. Furthmore, we filtered the candidate small indels by criteria: minimum depth (DP) is 8 and allele number (AN) is 4. We filtered SNVs by criteria: minimum depth (DP) for each allele in per sample is 10, allele number (AN) is 4, minimum base quality is 30, minimum qual by depth (QD) is 5, maximum mapping quality zero (MQ0) is 4, removing SNVs that were located on indel regions or in SNV cluster regions (defined by 3 SNV calling in a 10 bp window).

DELLY was used to detect structural variants from discordantly mapped read pairs [86]. The predicted SVs were classified as four groups: deletions, Inversions, tandem duplications and translocations. To avoid false-positive SVs in GK, DELLY was run with “-p” option that combined discordant alignment with split-read to get higher confident SVs. Then GK SVs were compared with Wistar SVs to get GK/Slac specific SVs.

The software BIC-seq [87] was used to detect copy number alterations between GK strain and Wistar strain. Differential CNVs were selected by two criteria: a) ratio of mapped reads number between GK and Wistar is greater than 2; b) Bofferoni adjusted p-value is smaller than 0.01.

### Variant density and function annotation

To illustrate the genome-scale difference between GK/Slac and Wistar/Slac, we analyzed the density and distribution of GK/Slac specific variants. Reference genome were segmented into 1Mb bins and variant density was defined as the number of variants in each bin divided by the number of nucleotide bases in the same bin that were covered by at least three reads in GK sequencing.

ANNOVAR was used to annotate SNVs/indels to gene region (exonic, splicing, ncRNA, UTR, intronic, up/down-stream, and intergenic). Functional impacts of exonic SNVs/indels were further classified as synonymous, nonsynonymous, stopgain, stoploss, and frameshift indels. SIFT was used to predict whether a nonsynonymous SNV affects protein function. SVs and CNVs were compared with gene annotation to get effected genes.

### Identification of potential T2D candidate genes

Genotype was encoded as allele values separated by “/”. The allele values are 0 for the reference allele (what is in BN rat), 1 for the first variant allele, 2 for the second variant allele and so on. We compared SNVs and indels called from GK/Slac and Wistar/Slac, chose GK/Slac strain specific variants that are not presented or differently presented in Wistar/Slac strain. These GK/Slac specific variants were classified into five groups based on their genotypes in GK/Slac and Wistar/Slac: 1) 0/1, 0/0; 2)1/1, 0/0; 3)1/1, 0/1; 4)1/2, 0/0; 5)1/2, 0/1.

Further analysis focused on protein affecting variants (PAV): nonsynonymous, stopgain, stoploss, frameshift indels, splicing, and exonic ncRNA. We investigated their genotype profiles in 28 sequenced rat strains (including 1 GK strain and multiple Wistar strains arising from the different geographical locations), whose genomes were sequenced by Atanur et.al. [46] and downloaded from RGD database. Whole-genome SNPs in GK/Slac, Wistar/Slac and 28 sequenced rat strains were compared. Distance between all possible pairs of strains were measured by net nucleotide substitutions [88]. The phylogenetic tree was constructed using UPGMA (unweighted pair-group method with arithmetic means) method in MEGA 6.06 package [89]. T2D related candidate PAVs were selected if they were homozygous-variant in two GK strains (GK/Slac sequenced in our experiment, GK sequenced by Atanur.et.al.) and was not homozygous-variant in eleven Wistar-derived strains (SHR/NHsd, SHRSP/Gla, SHR/OlaIpcv, WKY/NCrl, WKY/Gla, WKY/NHsd, LEW/Crl, LEW/NcrlBR, WAG/Rij, MHS/Gib, MNS/Gib).

### Functional analysis of potential candidate genes

Candidate gene lists were further filtered by integrating other information: T2D prior genes, protein-protein interaction, and differential gene expression.

We manually curated T2D related genes from published literatures and human GWAS catalog (http://www.genome.gov/admin/gwascatalog.txt) [90]. Totally, 506 T2D related genes were collected as prior genes (S6 File), including T2D susceptible genes, genes involved in important T2D pathways (such as insulin signaling pathway, adipocytokine signaling pathway), and genes associated with T2D related traits. Genes with GK/Slac specific variants were compared with T2D prior genes to narrow down the candidate gene list.

Known and predicted protein-protein interaction were obtained from STRING database [91], which quantitatively integrates interaction data from previous knowledge, genomic context, high-throughput experiments and conserved gene co-expression. We only used interaction pairs whose score is higher than 0.4. For T2D candidate gene list, we counted the number of their interaction partners in rat genome and in 506 T2D prior genes. Then we used Fisher test to calculate P-value, and adjusted it by multiple test. Genes with P-value< = 0.05 were regarded as better candidate genes.

Gene expression data was downloaded from a GEO dataset GSE13271(http://www.ncbi.nlm.nih.gov/geo/query/acc.cgi?acc=GSE13271), which measured the expression of GK and Wistar in three tissues (liver, muscle and adipose) at five time points during T2D development [92]. To compare GK and Wistar, T-test and fold-change threshold was used to get significantly differentially expressed genes (P-value< = 0.01 and fold-change>2); R package DCGL 2.0 [93] was used to mine differentially co-expressed genes (P-value< = 0.05 after Bonferroni correction). Results of each time point were combined to get the final differential gene list.

## Supporting Information

S1 File Detail of four libraries for whole genome sequencing of GK/Slac and Wistar/Slac rats. A total of 100.4 Gb (35.9X) and 85.6 Gb (30.5X) of short reads were generated for GK/Slac (Wistar/Slac) rat, respectively.
(XLS)

S2 File Statistics for quality control and genome mapping results. (XLS)

S3 File Statistics for post-mapping quality control. Number of filtered reads were shown in the table. Values in parentheses are the percentage of filtered reads in total mapping results.
(XLS)

S4 File Summary of variants in GK and Wistar rat, with BN rat as reference genome. (XLS)

S5 File Chromosome segments which have higher (top 5%) or lower (bottom 5%) SNV density and indel density. Density was defined as the number of SNV/indel in each 1Mb bin divided by the number of nucleotide bases in the same bin that were covered by at least three reads in GK sequencing. Low-density regions overlapped with gap were removed.
(XLS)

S6 File A list of T2D prior genes that were collected from literatures or human GWAS catalog. (XLS)

S7 File GK/Slac specific variants and functional effect on genes. (A) Protein affecting SNV (B) Protein affecting indels. (C) Structural variations that overlapped with gene. (D) Copy number gain or loss that overlapped with gene.
(XLS)

S8 File Progress for selecting potential T2D candidate genes. (A) 300 GK/Slac specific PAVs in 252 genes, which are homozygous mutant locus in GK/Slac strain but not in Wistar derived strains [46]. (B) After removing 60 ORs genes, there are 228 GK/Slac specific PAVs in 192 genes. These genes are analyzed by the following steps: 1) comparison with T2D prior genes; 2) differential (co-)expression between GK and Wistar rats in liver, muscle or adipose; 3) protein-protein interaction with T2D prior genes.
(XLS)
